# Societal activities associated with SARS-CoV-2 infection: a case-control study in Denmark, November 2020

**DOI:** 10.1017/S0950268821002478

**Published:** 2021-11-17

**Authors:** Pernille Kold Munch, Laura Espenhain, Christian Holm Hansen, Luise Müller, Tyra Grove Krause, Steen Ethelberg

**Affiliations:** 1 Department of Infectious Disease Epidemiology and Prevention, Statens Serum Institut, 5 Artillerivej, 2300, Copenhagen S, Denmark; 2 Division of Infectious Disease Preparedness, Statens Serum Institut, 5 Artillerivej, 2300, Copenhagen S, Denmark; 3 Department of Public Health, Global Health Section, University of Copenhagen, Øster Farimagsgade 5, 1014, Copenhagen K, Denmark

**Keywords:** Case-control Study, Community acquired infections, COVID-19, Risk factors, SARS-CoV-2

## Abstract

Identification of societal activities associated with SARS-CoV-2 infection may provide an evidence base for implementing preventive measures. Here, we investigated potential determinants for infection in Denmark in a situation where society was only partially open. We conducted a national matched case-control study. Cases were recent RT-PCR test-positives, while controls, individually matched on age, sex and residence, had not previously tested positive for SARS-CoV-2. Questions concerned person contact and community exposures. Telephone interviews were performed over a 7-day period in December 2020. We included 300 cases and 317 controls and determined odds ratios (ORs) and 95% confidence intervals (95% CI) by conditional logistical regression with adjustment for household size and country of origin. Contact (OR 4.9, 95% CI 2.4–10) and *close contact* (OR 13, 95% CI 6.7–25) with a person with a known SARS-CoV-2 infection were main determinants. Contact most often took place in the household or work place. Community determinants included events with singing (OR 2.1, 95% CI 1.1–4.1), attending fitness centres (OR 1.8, 95% CI 1.1–2.8) and consumption of alcohol in a bar (OR 10, 95% CI 1.5–65). Other community exposures appeared not to be associated with infection, these included shopping at supermarkets, travel by public transport, dining at restaurants and private social events with few participants. Overall, the restrictions in place at the time of the study appeared to be sufficient to reduce transmission of disease in the public space, which instead largely took place following direct exposures to people with known SARS-CoV-2 infections.

## Introduction

COVID-19, caused by novel coronavirus SARS-CoV-2, was declared a pandemic by the World Health Organization (WHO) on 11 March 2020 [1]. SARS-CoV-2 has since been confirmed in more than 200 countries with more than 180 million confirmed cases and almost 4 million confirmed deaths as of 30 June 2021 [2].

In Denmark, a national comprehensive lockdown was imposed on 13 March 2020, a few weeks after the first diagnosed case appeared [3–5]. Since then, national and regional measures and restrictions have been introduced and gradually lifted and reintroduced following the development of the epidemic. Such measures and restrictions aim to reduce the number of contacts and have included limiting public gathering, encouraging working from home, closures of schools and higher education facilities, closure of public spaces and hospitality venues (e.g. restaurants, cultural and sports facilities) and mandatory mask use [6, 7]. While several measures target community exposures, their importance for transmission of disease is not well understood. The effect of the individual measures and restrictions on transmission of SARS-CoV-2 is not easily measured and there is a need for more knowledge about the relative contribution of the various sources of community exposures in general so that target measures can be implemented correctly.

Transmission within households and between family members is reported to play an important role in SARS-CoV-2 transmission [8–10]. Other risk factors reported from previous studies include travelling by public transport [11, 12] and airplane [13], working in frontline service work [14], working or studying away from home [13] as well as socio-economic determinants such as lower educational level [11], maintainance income, unemployment, low household income, ethnicity [14, 15] and living in social housing [16]. A case-control study among outpatients in the USA found that SARS-CoV-2-positive cases were more likely to have been dining at restaurants and visiting bars/coffee shops than SARS-CoV-2-negative controls. The study indicated no increased risk associated with shopping, visiting friends and family and use of public transport [17]. Another case-control study among French adults found a risk associated with several community exposures including attending public or private gatherings, bar and restaurant visits, and indoor sport activities [18].

Here, we aimed to identify societal activities associated with SARS-CoV-2 infection in Denmark in the adult population. The study was performed early December 2020, in a situation where a number of societal restrictions were imposed and while wild-type SARS-CoV-2 (Wuhan variant) was the predominant strain in circulation and before vaccinations had begun.

## Methods

### Study design and participants

We conducted a national individually matched case-control study to assess associations of community and contact exposures with SARS-CoV-2 infection in the adult population. In the study period, free-of-cost RT-PCR tests were offered to all Danes from public test stations [19]. All test results were stored in a person identifiable format in a national database [20], from where eligible cases were extracted. Eligible cases were persons residing in Denmark aged 18–65 years with domestically acquired, RT-PCR-confirmed SARS-CoV-2 infection with a sample date in the period from 4 to 6 December 2020 (both days included). We aimed to include one matched control person per case. Controls were matched to cases by one year age intervals, sex (two levels) and municipality (98 levels) and were extracted from the Danish Civil Registration System [21]. Cases and controls were interviewed via telephone from 9 to 15 December by a sub-contracted polling company. Cases and controls were excluded if they had stayed overnight outside of Denmark, had been hospitalised for more than 12 h in the period of interest, or were unable to complete the interview, e.g. because of limited Danish language skills. Controls were excluded if they had an RT-PCR-confirmed SARS-CoV-2 infection recorded in the national laboratory database at any time prior to 8 December 2020, or reported at the interview that they had tested SARS-CoV-2-positive by RT-PCR at any time prior to the interview.

### Exposures included in the questionnaire

A short tailored questionnaire was developed and set up using a web-based survey tool. Trained interviewers carried out the inclusion procedure and interviews via telephone. The questionnaire included questions on a range of contact and community exposures in the 14-day period prior to symptom onset or test date for cases or the equivalent 14-day period for their matched controls. Contact exposures included *close contact/other contact* with a person with known SARS-CoV-2 infection. We used the *close contact* definition set by Danish authorities: exposure to a household member, direct physical contact (e.g. hugging), unprotected and direct contact with secretions from an infected person, having been within a distance of <1 m for more than 15 min, or caring for COVID-19 patients where the prescribed protective equipment had not been used. *Other contact* was defined as contact with a person with known SARS-CoV-2 infection. Further, we collected information on number of contacts during work or education, and the number of close contacts (10 or less, 11–20, 21–50, or more than 50).

Community exposures included activities as dining at restaurants, going to bars, shopping, participating in sport activities, and religious events and events with singing, etc., along with question on if this took place indoors or outdoors, or involved consumption of alcohol. Cases were additionally asked about suspected place of infection, symptoms and symptom onset. Questions on community exposures could generally be answered on a scale from ‘never’, ‘one-two times’, ‘three or more times’ (for public transport ‘never’, ‘one-two times’, ‘three-six times’ or ‘seven or more times’; for private social events: ‘less than 10’, ‘10–20’ or ‘more than 20 people’). Lastly, we collected information about the degree to which recommendations concerning prevention of infection were followed; these included washing hands and wearing face mask with response options ranging from always to never (four categories). From the Danish Civil Registration System, we collected data on country of origin (three levels: Danish, first or second generation western, or non-western, immigrant) and household size (number of registered persons on the same address, five levels: 1, 2, 3, 4, ≥5 persons).

### Power calculation and data collection

The required sample size was calculated based on an expected restaurant visit frequency of 10% among controls [22]. With a power of 80%, an *α*-level of 0.05 and an odds ratio to detect at 2, we needed 566 participants following standard sample size formulae for unmatched case-control studies [23]. We aimed to include 600 (300 cases and 300 controls). For cases, we expected a 20% inclusion rate, and therefore randomly extracted 1500 cases (out of 3919 persons testing positive during the four days).

We aimed to include the first 300 cases with a valid publicly available phone number. At least two attempts were made to call each eligible case and control each day. Matched controls were contacted after a case interview had been completed.

### Data analysis

We compared basic demographic characteristics (age, sex, region and country of origin) of included cases with all confirmed cases from the population using Pearson's *χ*^2^ test. We compared exposures reported by cases with those of controls using conditional logistic regression. For second level questions that were only asked to participants answering yes to overall questions, matched analyses could not be performed and instead adjustment for the matching variables was performed using logistic regression. Throughout, we present matched odds ratios (mOR) or odds ratios adjusted for the matching variables (OR) with 95% confidence intervals (95% CI). All analyses were furthermore adjusted for country of origin and household size.

For the analyses, answers to community exposures were dichotomised as ‘never’ *vs.* ‘one or more times’ during the 14 days before illness onset. To avoid diluting a potential association with community exposures, we excluded cases (and their matched controls) who reported to be infected in their household. Statistical analyses were conducted using STATA version 14.2.

### COVID-19 measures and restrictions in place, November 2020

During the study period, the following restrictions were imposed. The maximum number of people allowed to gather publicly was 10; the same was recommended in private homes. Further, citizens were recommended to have a maximum of 10 close contacts. The maximum number of people who could gather was also applicable at sport facilities, restaurants, etc. Where possible, citizens were recommended to work from home.

Restaurants, cafes, bar, etc., had to close at 10 pm and it was mandatory to wear a face mask indoors, except when seated. Discotheques and nightclubs were closed. In retail shops, face mask or face shields were mandatory and no sale of alcohol was allowed after 10 pm. At professional sport events, a maximum of 500 seated people could gather (facing the same direction). The same was applicable for religious events and cultural events. At all other indoor public places, face masks were mandatory (public transport, cultural events, sport facilities, health care setting, etc.) [24].

To prevent the spread of SARS-CoV-2, the Danish Health Authority recommended to (1) stay home if having symptoms of COVID-19, (2) wash hands often and use hand sanitiser, (3) cough or sneeze in the sleeve, (4) limit physical contact, (5) clean indoor areas thoroughly, and (6) keep 1 m distance and ask others to be considerate [25].

## Results

**Fig. 1.**
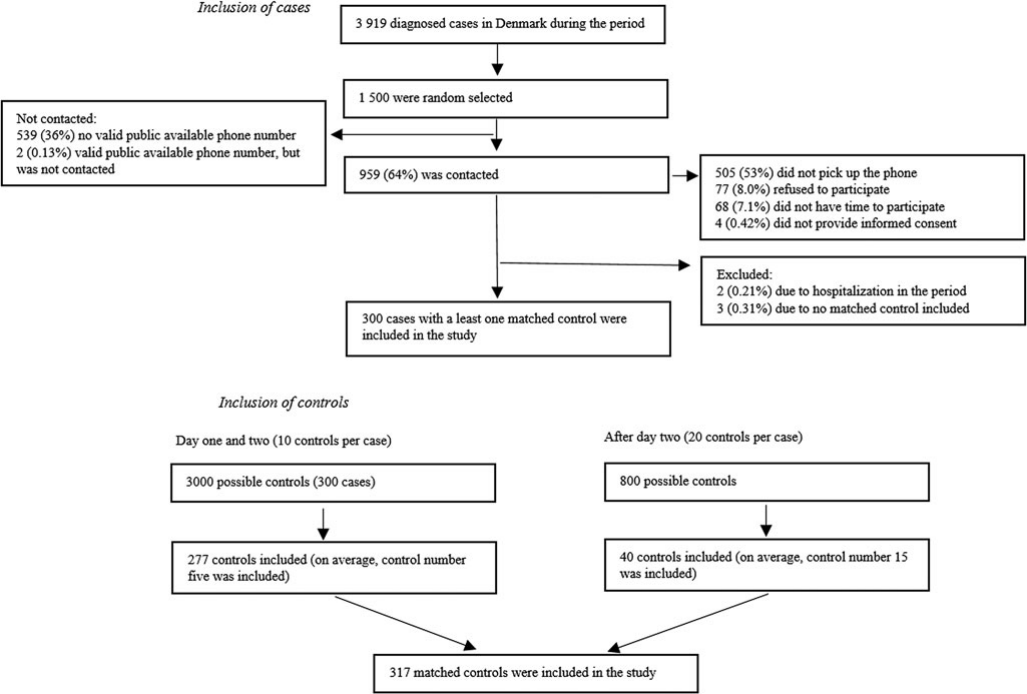
Flow diagram depicting inclusion of cases and control during data collection.

A total of 300 cases and 317 matched control persons were included in the study (Fig. 1). There was no difference in country of origin, employment and household size between cases and controls (Table 1). Of the 1 500 extracted cases, 961 (64%) had a valid publicly available phone number. Of those, 77 (8.0%) cases refused to participate, 505 (53%) did not pick up the phone, 68 (7.1%) did not have time to participate. Cases in the age group of 18–29 years and with a country of origin different from Danish were overrepresented among people for whom no valid phone number could be found (Table 1). Cases included in the study were older and more likely to be of Danish origin, compared to all who tested positive for SARS-CoV-2 in the period (Table 1).

**Table 1.** Number and proportion of all persons who tested RT-PCR-positive for SARS-CoV-2 in Denmark in the period (4–6 December 2020), included cases and controls by demographic characteristics and *P* value of test for deviations

Demographic characteristics	RT-PCR-positive (*n* = 3919), *n* (%)	Included cases (*n* = 300), *n* (%)	*P* value^a^	Matched controls (*n* = 317), *n* (%)	*P* value^b^
*Age group*			<0.05		
18–29 years	1346 (34)	82 (27)		86 (27)	
30–44 years	1140 (29)	75 (25)		80 (25)	
45–59 years	1183 (30)	110 (37)		113 (36)	
60–65 years	250 (6.4)	33 (11)		38 (12)	
*Sex*			0.367		
Male	1906 (49)	154 (51)		166 (52)	
Female	2013 (51)	146 (49)		151 (48)	
*Region*			0.172		
Capital Region of Denmark	2229 (57)	149 (50)		155 (49)	
Region Zealand	508 (13)	44 (15)		47 (15)	
Region of Southern Denmark	386 (9.8)	32 (11)		36 (11)	
Central Denmark Region	665 (17)	62 (21)		65 (21)	
North Denmark Region	131 (3.3)	13 (4.3)		14 (4.4)	
*Country of origin*			<0.05		0.130
Danish	2992 (76)	256 (85)		285 (90)	
First or second generation western	253 (6.5)	17 (5.7)		15 (4.7)	
Non-western immigrant	674 (17)	27 (9)		16 (5.1)	
Missing	–	–		1 (–)	
*Employment*^c^					0.104
Employed		216 (72)		235 (74)	
Under education		51 (17)		36 (11)	
Retired		10 (3.3)		19 (6.0)	
Other		22 (7.4)		27 (8.5)	
Missing		1 (–)		–	
*Household size*^c^^.^^d^					0.108
1		30 (10)		50 (16)	
2		98 (33)		87 (27)	
3		63 (21)		69 (22)	
4		75 (25)		86 (27)	
≥5		34 (11)		25 (7.9)	

a Difference between persons who tested RT-PCR-positive for SARS-CoV-2 and included cases.

b Difference between included cases and controls.

c Details were only available among participants.

d Number of registered persons on the same address.

In total, 90% of cases reported a likely place of infection. Such exposure was primarily reported to have happened at the workplace (30%), in the household (29%) or among friends (13%). Cases further reported education facilities (3.8%), leisure activities (3.8%), other events (1.6%), or ‘other place/exposure’ (8.6%) as likely places of infection (Supplementary Materials, Table S1). When inquired about the presence or absence of symptoms, 88% of cases reported having experienced COVID-19 symptoms (Supplementary Materials, Table S1). Cases and controls were queried about contact with other persons with known SARS-CoV-2 infection (Table 2). Among cases, the odds of having been in *close contact* with an infected person were 13 times higher than for controls. For cases specifically reporting *close contact*, 41% had had this in their households. *Other contact* to a person with known SARS-CoV-2 infection also carried increased odds (OR 4.9, 95% CI 2.4–10) (Table 2). Disregarding cases who were infected at home, cases who had a job were more likely than controls who had a job to report being in contact with many different people (≥50 persons) at work (OR 1.7, 95% CI 1.1–2.6). We observed no difference in number of close contacts between cases and controls where three out of four had 10 or fewer close contacts during the 14-day period (Table 2).

**Table 2.** Number, proportion and odds ratio related to contact with a person with known SARS-CoV-2 infection and number of contacts, Denmark, November 2020

Items (*n* cases/*n* controls)	Cases (*n* = 300), *n* (%)	Controls (*n* = 317), *n* (%)	OR (95% CI)
*Close contact*	124 (41)	22 (6.9)	13 (6.7-25)^a^
*Other contact*	164 (55)	39 (12)	4.9 (2.4-10)^b^
*Close contact in the household (122/22)*^c^	50 (41)	3 (14)	5.03 (1.26-20)^d^
*More than 10 close contacts (208/221)*^e^	55 (26)	56 (25)	1.08 (0.68-1.73)^f^
Number of close contacts (208/221)^e^			
0–10	153 (74)	165 (75)	1 (ref.)
11–20	33 (16)	38 (17)	1.04 (0.60-1.83)^f^
21–50	14 (6.7)	15 (6.8)	0.80 (0.35-1.83)^f^
>50	8 (3.8)	3 (1.4)	3.00 (0.77-11.74)^f^
*Contact with many different people during work (148/235)*^g^	58 (39)	67 (29)	1.66 (1.06-2.60)^f^
Contact during work (148/235)			
No contact (working from home, alone at the office, etc.)	14 (9.5)	37 (16)	1 (ref.)
Same people - not more than 50 different people (office, etc.)	73 (49)	128 (54)	1.61 (0.79-3.27)^d^
Many different people (working within the healthcare, shops, etc.)	58 (39)	67 (29)	2.48 (1.19-5.17)^d^

a mOR adjusted for other contact, country of origin and household size.

b mOR adjusted for close contact, country of origin ad household size.

c Close contact with a person with known COVID-19 in the household *vs.* close contact with a person with known COVID-19 other than in the household.

d OR adjusted for sex, age, region, country of origin and household size.

e Analysis performed without household transmission.

f mOR adjusted for country of origin and household size.

g Contact with many different people *vs.* contact with the same, no contact or other during work.

For the analyses of exposures taking place in the community, we excluded cases reporting likely household transmission and their matched control persons. Cases were more likely than control persons to report attending events where they or others were singing (OR 2.1, 95% CI 1.1–4.1) and having been in a fitness centre (OR 1.8, 95% CI 1.1–2.8) (Table 3). No difference was observed for participation in other indoor or outdoor sports activities. Participating in private social events with several participants, religious events, sport events as spectators, visiting bars or attending indoor cultural events were positively but non-significantly associated with SARS-CoV-2 infection (Table 3). For visits to bars, an association was found when asked if alcohol consumption was involved (OR 10, 95% CI 1.5–65). In contrast, controls more often than cases reported having been to restaurants (OR 0.6, 95% CI 0.4–1.0) and shops other than grocery shops (OR 0.6, 95% CI 0.4–1.0).

**Table 3.** Number, proportion and odds ratios related to community exposures and protective behaviour without household transmission, Denmark, November 2020

Community exposures (*n* cases/*n* controls)^a^	Cases (*n* = 208), *n* (%)	Controls (*n* = 221), *n* (%)	OR (95% CI)
*Restaurant*	79 (38)	107 (48)	0.64 (0.43–0.96)^b^
Indoor *vs.* outdoor (79/156)	74 (94)	150 (96)	0.75 (0.20–2.77)^c^
Evening *vs.* day (79/98)	59 (75)	98 (63)	1.79 (0.94–3.43)^c^
Alcohol *vs.* no alcohol (79/156)	6 (7.6)	12 (7.7)	0.85 (0.28–2.57)^c^
*Bar*	35 (17)	29 (13)	1.42 (0.82–2.46)^b^
Evening *vs.* day (35/40)	32 (91)	37 (93)	4.53 (0.21–99)^c^
Alcohol *vs.* no alcohol (35/40)	26 (74)	24 (60)	10 (1.53–65)^c^
*Indoor cultural events*	47 (23)	41 (19)	1.36 (0.85–2.18)^b^
Alcohol *vs.* no alcohol (47/57)	6 (13)	6 (11)	0.61 (0.13–2.79)^c^
*Spectator at sport events*	9 (4.3)	8 (3.6)	1.74 (0.54–5.63)^b^
*Indoor fitness centre*	60 (29)	44 (20)	1.77 (1.11–2.84)^b^
*Indoor sport activities*	24 (12)	22 (10)	1.05 (0.55–2.03)^b^
*Outdoor sport activities*	15 (7.2)	21 (10)	0.79 (0.38–1.65)^b^
*Shopping (grocery)*	192 (92)	210 (95)	0.62 (0.28–1.38)^b^
*Shopping (other)*	124 (60)	155 (70)	0.64 (0.43–0.95)^b^
*Private social events <10 persons*	79 (38)	99 (45)	0.80 (0.53–1.21)^b^
Alcohol *vs.* no alcohol (79/140)	22 (28)	33 (24)	0.97 (0.47–2.02)^c^
Adheres to the official guidelines (79/140)	38 (48)	71 (51)	0.91 (0.49–1.68)^c^
*Private social events 10–20 persons*	11 (5.3)	17 (7.7)	0.64 (0.28–1.45)^b^
*Private social events >20 persons*	6 (2.9)	2 (0.90)	3.27 (0.65–17)^b^
*Public transport*	75 (36)	82 (37)	1.00 (0.63–1.57)^b^
Rush hours (75/123)	28 (37)	45 (37)	1.03 (0.51–2.06)^c^
*Religious events*	17 (8.2)	9 (4.1)	1.99 (0.85–4.65)^b^
*Events with singing*	29 (14)	19 (8.6)	2.08 (1.06–4.08)^b^
Prevention of infection^d^			
Good hand hygiene	204 (98)	219 (99)	1.98 (0.35–11)^b^
Cough or sneeze into sleeve	207 (100)	218 (99)	0.45 (0.04–4.51)^b^
Cleaning	187 (90)	192 (87)	0.80 (0.43–1.48)^b^
Keeps minimum 1 m distance	193 (93)	206 (93)	1.11 (0.50–2.44)^b^
Avoid places with many people	191 (92)	196 (89)	0.69 (0.36–1.34)^b^
Minimise activities where you have contact with other people	189 (91)	203 (92)	0.93 (0.45–1.92)^b^
Wear face mask when required	206 (99)	216 (98)	0.44 (0.08–2.36)^b^

For each exposure, the distribution for cases and controls and the mOR are shown without household transmission. For the detailed conditions, all controls are included, and the ORs are shown.

a Never *vs.* at least once in the 14 days prior to illness onset.

b mOR adjusted for country of origin and household size.

c OR adjusted for sex, age, region, country of origin and household size.

d Always or often versus rarely or never.

We observed no difference among cases and controls in their reported compliance concerning: good hand hygiene, wearing masks when required, frequent cleaning at home, keeping minimum 1m distance to other people, avoiding places with many other people, minimising/limiting activities involving contact with other people and showing good cough and sneeze etiquette. Compliance with imposed measures and hygiene advice was generally indicated by more than 90% of the participants (Table 3).

## Discussion

In this national case-control study of determinants associated with SARS-CoV-2 infection, we found that having had contact, in particular *close contact*, to another person with a known SARS-CoV-2 infection was strongly associated with infection. Community exposures associated with infection were participation in events where people sang, fitness centres and consumption of alcohol in a bar. Other community exposures appeared not to be associated with infection, e.g. supermarkets, public transport and restaurants.

Other studies have also found that contact with a known person infected with SARS-CoV-2 is the main risk factor for infection [10, 13, 17, 26]. Further, we found that cases were more likely to have had close contact within the household, where controls were more likely to have close contact at work. Household transmission appears to play an import role in the transmission of SARS-CoV-2 infection [8–10]. As reported elsewhere, the risk of transmission seemed to be higher if the infected close contact showed symptoms of COVID-19 [8]. Outside of the household, having a job involving contact with many different people during the day was associated with an increased risk, a finding that has also been reported by others [13]. Different job types have previously been found to be associated with COVID-19. In Denmark, mink workers have been found to have high occupational risk [27] and another study found that health care workers had higher levels of antibodies against SARS-CoV-2 than blood donors [28], while researchers in Norway found different patterns in occupational risk in the first and the second wave of infection [29]. In France, office work without teleworking was found to increase the risk of SARS-CoV-2 [18].

In this study, we investigated community exposures in a situation where a number of societal restrictions were already in place [24] and where vaccines had not yet been licensed and introduced in Denmark [30]. Overall, we found few determinants for community transmission. This may indicate that the rules and restrictions that were in place at the time were effective, although most activities were merely restricted rather than fully closed-down. In support of this, we found that the vast majority of both cases and controls reported to comply with restrictions and official advice. In other surveys from 2020, Danes have been found to trust health authorities and to show high degrees of compliance with the national policy to limit the transmission of COVID-19 [31, 32]. Use of a fitness centre was found to be associated with SARS-CoV-2 infection; this was also one of only few activities found as risk factors in another (unpublished) Danish study [33]. This finding may be expected, as a large number of individuals might be present with deep breathing and excretion of aerosols, close interactions might occur, the likelihood of touching the same infected items might be high and recycled air might increase the risk of transmission compared to outdoor activities. In line with this, and a systematic review [34], we found no association with outdoor sports activities and SARS-CoV-2 infection. However, contrary to findings from France, we saw no association with other indoor sports activities [18]. More focused studies are needed in order to determine which exposures – for instance type of training, length of work out, ventilation, etc. – in fitness centres play a role in the association with COVID-19.

We found having participated in events involving singing to be associated with SARS-CoV-2 infection. Participation in choirs or similar group singing activities has been banned in some countries due to high infection rates and outbreaks and the airflow pattern during singing has been investigated [35]. Consumption of alcohol at bars was found to be associated with SARS-CoV-2. A recent study examined the behaviour of consumers and staff at bars upon the re-opening. Despite the rules and guidance, the authors observed close physical interaction especially when customers were drinking, which might explain the association [36–38]. Eating at restaurants, on the other hand, was not found to be associated with infection. At the time of the study, restaurants had to close at 10 pm, with a minimum distance of 2 m between guests from separate parties and a requirement for guests to wear masks when not seated. With this study, we were not able to identify activities that would have been associated with COVID-19 in a situation without restriction in place, but could only identify any residual risk associated with activities under the given restrictions. Nevertheless, the mentioned restrictions appear to have been adequate to prevent transmission under the circumstances. Shortly after this study was performed, additional restrictions were introduced, including a closure of fitness centres, bars were shut down, and restaurants became open for takeaway sales only. In that situation, it would not have been possible to study if these societal activities were associated with infection.

A methodological strength of the study was the random sampling of cases and matched controls, followed by a matched statistical analysis, which minimises selection bias. Denmark had one of Europe's highest test capacities with easy and free access to PCR test, and all close contacts were offered testing at day 4 and 6 after exposure [19]. This reduces the risk of including undiagnosed cases as controls. Further, we were able to adjust for country of origin and household size through access to public registers. Telephone interviews done over a short period of time will likely result in more precise and complete answers compared to a set-up using postal questionnaires. We were also able to exclude cases likely infected in the household when doing the community exposure analysis.

Limitations to the study include the sample size. Some community exposures were vaguely associated with SARS-CoV-2 infection, without reaching statistical significance. It is possible that these would have appeared as risk factors, if our study had had more power. These factors include attending private social events with several participants, religious events, sport events as spectator, visiting bars and attending indoor cultural events. Other potential limitations include a potential recall bias for self-reported contact and community exposures, if cases had better recollection than controls because they would have had time to consider the circumstances of the exposure. Additionally, selection bias may have affected the applicability of the results, if those who have a publicly available phone number choose to answer the phone and agree to participate had a different behaviour than those who did not. This might potentially explain the high degree of compliance with imposed measures and hygiene advice amongst the participants. For cases, we saw that non-western immigrants were underrepresented compared to the source population of all RT-PCR-confirmed cases at the time, and our results may not be applicable to this population. Potential systematic differences between cases and controls might work to both under- or overestimate the calculated odds ratios. For contact exposures, the participants were asked if they had had any contact with an infected person in the period of interest. This was defined by the participant themselves, and not the authority responsible for contact tracing. It should also be noted that the study was performed before vaccinations began and at a time when wild-type SARS-CoV-2 variants circulated in Denmark, i.e. before the introduction of the *α*-variant [39–41]. It is possible that circulation of more infectious variants would have challenged the community restrictions that were in place when the study was performed.

In conclusion, this study was done in period of time where a number of partial restrictions were in place. It may appear that they have been efficient, since the majority of transmission took place via family members and colleagues. Several community exposures appeared not to be associated with any risk under the given restrictions, such as shopping in supermarkets, using public transport and going to restaurants, while singing, consuming alcohol and attending fitness centres were associated with increased transmission. These findings are useful when evaluating the COVID-19 measures and for planning potential future measures aiming to restrict epidemic disease transmission.

## Data Availability

The collected data from this study may be made available from the corresponding authors upon reasonable request. Sources of publicly searchable databases are indicated in the introduction.

## Supplementary material

For supplementary material accompanying this paper visit https://doi.org/10.1017/S0950268821002478. click here to view supplementary material

## Code availability

Statistical analyses were conducted using STATA version 14.2.
