# Supplementary material for: Societal activities associated with SARS-CoV-2 infection: a case-control study in Denmark, November 2020
Source: Epidemiol Infect. 2021 Nov 17;150:e9. doi: 10.1017/S0950268821002478 (PMC8755547; doi:10.1017/S0950268821002478)
Supplement: Supplementary file 1 [file S0950268821002478sup001.docx]

**Supplementary Material**

*Epidemiology and Infection*

Societal activities associated with SARS-CoV-2 infection – a case-control study in Denmark, November 2020

Pernille Kold Munch, Laura Espenhain, Christian Holm Hansen, Luise Müller, Tyra Grove Krause and Steen Ethelberg

| **Table S1** Number and proportion of cases who experienced COVID-19 symptoms (n=300) and place of likely infection (n = 315) | |
| --- | --- |
|  | n, (%) |
| COVID-19 symptoms | 264 (88) |
| *Place of likely infection* | |
| Workplace | 95 (30) |
| Household | 92 (29) |
| Friends home | 40 (13) |
| Education facilities | 12 (3.8) |
| Leisure activity | 12 (3.8) |
| Other events | 5 (1.6) |
| Other place/exposure | 27 (8.9) |
| Unknown | 32 (10) |
